# A qualitative study exploring perceived barriers and enablers to fidelity of training and delivery for an intervention to reduce non-indicated imaging for low back pain

**DOI:** 10.1186/s12998-023-00480-6

**Published:** 2023-01-31

**Authors:** Daphne To, Diana De Carvalho, Andrea Pike, Rebecca Lawrence, Holly Etchegary, Andrea M. Patey, Elaine Toomey, Amanda Hall

**Affiliations:** 1 Division of Community Health and Humanities, Faculty of Medicine, Memorial University of Newfoundland, Saint John’s, NL A1B 3V6 Canada; 2 Primary Healthcare Research Unit, Faculty of Medicine, Memorial University of Newfoundland, Saint John’s, NL Canada; 3 Centre for Implementation Research, Ottawa Hospital Research Institute – General Campus, Ottawa, ON Canada; 4 School of Allied Health, Faculty of Education and Health Sciences, University of Limerick, Limerick, Ireland

**Keywords:** Diagnostic imaging, Evidence-based practice, Implementation science, Intervention fidelity, Low back pain, Needs assessment, Theoretical Domains Framework

## Abstract

**Background:**

Non-specific low back pain (LBP) commonly presents to primary care, where inappropriate use of imaging remains common despite guideline recommendations against its routine use. Little is known about strategies to enhance intervention fidelity (i.e., whether interventions were implemented as intended) for interventions developed to reduce non-indicated imaging for LBP.

**Objectives:**

We aim to inform the development of an intervention to reduce non-indicated imaging among general practitioners (GPs) and chiropractors in Newfoundland and Labrador (NL), Canada. The study objectives are: [1] To explore perceived barriers and enablers to enhancing fidelity of training of GPs and chiropractors to deliver a proposed intervention to reduce non-indicated imaging for LBP and [2] To explore perceived barriers and enablers to enhancing fidelity of delivery of the proposed intervention.

**Methods:**

An exploratory, qualitative study was conducted with GPs and chiropractors in NL. The interview guide was informed by the National Institutes of Health Behavior Change Consortium fidelity checklist; data analysis was guided by the Theoretical Domains Framework (TDF). Participant quotes were coded into TDF domains, belief statements were generated at each domain, and domains relevant to enhancing fidelity of provider training or intervention delivery were identified.

**Results:**

The study included five GPs and five chiropractors from urban and rural settings. Barriers and enablers to enhancing fidelity to provider training related to seven TDF domains: [1] Beliefs about capabilities, [2] Optimism, [3] Reinforcement, [4] Memory, attention, and decision processes, [5] Environmental context and resources, [6] Emotion, and [7] Behavioural regulation. Barriers and enablers to enhancing fidelity to intervention delivery related to seven TDF domains: [1] Beliefs about capabilities, [2] Optimism, [3] Goals, [4] Memory, attention, and decision processes, [5] Environmental context and resources, [6] Social influences, and [7] Behavioural regulation.

**Conclusion:**

The largest perceived barrier to attending training was time; perceived enablers were incentives and flexible training. Patient pressure, time, and established habits were perceived barriers to delivering the intervention as intended. Participants suggested enhancement strategies to improve their ability to deliver the intervention as intended, including reminders and check-ins with researchers. Most participants perceived intervention fidelity as important. These results may aid in the development of a more feasible and pragmatic intervention to reduce non-indicated imaging for GPs and chiropractors in NL.

**Supplementary Information:**

The online version contains supplementary material available at 10.1186/s12998-023-00480-6.

## Introduction

Non-specific low back pain (LBP) is a common condition [1] defined as LBP where the pathoanatomical cause of pain cannot be determined [2, 3]. Non-specific LBP likely develops from a complex interaction of biophysical, psychological, and social factors [4], and red flags indicative of specific spinal pathologies (e.g., fracture, infection, cancer), are typically not present in individuals who present with non-specific LBP in primary care [5]. Clinical practice guidelines for the management of LBP [6] recommend against the use of routine diagnostic imaging in patients with non-specific LBP, and most only recommend imaging in the presence of red flags or if imaging would change a patient’s treatment plan [6].

Despite relatively consistent guideline recommendations from around the world, the use of diagnostic imaging in primary care practices remains common [7, 8]. Various interventions have been developed to improve the appropriate use of imaging for LBP, including education interventions for clinicians, audit and feedback, and clinical decision support tools [9, 10]. However, the evidence of effectiveness for these interventions has been variable [9, 10]. One reason for the variation in effectiveness across studies may be due to poor intervention fidelity, meaning that interventions may not have been delivered or implemented as intended [11, 12].

In health behaviour change research, intervention fidelity refers to “the methodological strategies used to monitor and enhance the reliability and validity of behavioural interventions” [13, 14]. Knowledge of intervention fidelity can aid in the interpretation of the results of effectiveness trials [14]. For example, if an intervention was found to be effective but implemented with low fidelity, the effectiveness results may have been due to unknown factors added to or omitted from the intervention. If an intervention was found to be ineffective and was also implemented with low fidelity, it would not be possible to determine if the intervention was truly ineffective, or if it was just not implemented as intended.

The National Institutes of Health Behavior Change Consortium (NIHBCC) developed a framework for intervention fidelity, which includes five areas of fidelity: study design, training, delivery, receipt, and enactment [14]. Fidelity to study design refers to the study being able to adequately test the hypothesis in relation to an underlying theoretical framework. Fidelity to provider training refers to the training provided to the people who will be implementing an intervention. Fidelity to intervention delivery refers to delivering the intervention the way it was intended to be delivered by intervention developers. Fidelity to intervention receipt refers to the ability of participants to understand and perform the skills delivered during the intervention session. Fidelity to intervention enactment refers to the ability of participants to understand and perform the skills in real-life settings. The NIHBCC produced a validated checklist with strategies to enhance and/or assess intervention delivery within the five domains to accompany their intervention fidelity framework [13, 15]. The NIHBCC intervention fidelity framework has been applied in many studies of health behaviour change to assess the degree to which intervention fidelity has been reported, enhanced, and/or assessed [16–18].

A multi-jurisdictional project aiming to test the effectiveness of a theory-informed intervention to reduce non-indicated imaging for LBP is being planned. The intervention will be adapted from a similar intervention developed using the Behaviour Change Wheel and the Theoretical Domains Framework [19]. The intervention will consist of clinical education, a clinician-patient decision aid, and an educational booklet with reminders to indications for imaging and evidence-based, patient-specific management strategies. The intervention will first be implemented in Newfoundland and Labrador (NL), Canada. In the province of NL, medical practitioners (e.g., general practitioners) and chiropractors are able to order x-rays and regularly manage patients with LBP [20]. Based on a medical record review from GPs in NL, only 6.5% of referrals for lumbar spine CT imaging were considered appropriate (i.e., concordant with guideline or best practice recommendations) [21]. Among chiropractors in NL, a survey on their knowledge of and adherence to radiographic guidelines found that about half of respondents were unaware of or did not know current guideline recommendations for LBP radiography, and one quarter of respondents indicated they did not use guidelines to inform their clinical decisions [22]. Adherence, measured with clinical vignettes, ranged from 38 to 88% for not ordering an x-ray when it was not indicated [23].

Interventions which are well developed but poorly implemented are costly to patient care, health research, and health systems. While many interventions aimed at reducing non-indicated imaging for LBP have been developed, little attention has been paid to the degree to which these interventions have been implemented (i.e., intervention fidelity) [9, 24]. When developing new interventions, finding ways to enhance intervention fidelity in the early stages of intervention development can provide an opportunity to optimise the intervention and lead to a more accurate interpretation of the trial results [25, 26]. Healthcare professionals using interventions to aid in decision-making for the appropriate use of imaging for LBP require confidence in the effectiveness and implementation of the intervention they are using.

The overall aim of this study is to apply methods from implementation science to inform the design and deliverability of the previously described intervention to reduce non-indicated imaging for LBP in Newfoundland and Labrador, Canada.

This study had two objectives:

1. To explore barriers and enablers which were perceived to influence *fidelity of training* of general practitioners (GPs) and chiropractors to deliver a proposed intervention aimed at reducing non-indicated imaging for LBP.
2. To explore barriers and enablers which were perceived to influence *fidelity of delivery* of a proposed intervention aimed at reducing non-indicated imaging for LBP by GPs and chiropractors.

## Methods

### Design

We conducted an exploratory, qualitative study describing GPs’ and chiropractors’ perceived barriers and enablers to enhancing fidelity of training and delivery for a proposed intervention aimed at reducing non-indicated imaging for LBP. The perceived barriers and enablers were analysed using the Theoretical Domains Framework (TDF) [27]. A qualitative approach was chosen because it allowed the researchers to probe in greater detail about the proposed strategies to enhance fidelity of training and delivery, which would aid in the development of the overall intervention. This was particularly important since little is known about strategies to enhance intervention fidelity within the context of interventions to reduce the use of non-indicated imaging for LBP. This qualitative study was reported according to the COnsolidated criteria for Reporting Qualitative research (COREQ) checklist (Additional file 1). This study is part of a larger qualitative study that included multiple questions on enhancing and assessing fidelity to interventions to improve GP and chiropractor adherence to imaging guidelines for LBP; the full protocol and full interview guide for the larger study have been published [28]. The current study will focus on only the questions regarding the barriers and enablers to enhancing fidelity of training and delivery for these types of interventions.

### Participant selection

Community-based GPs and chiropractors who held a license and were registered in the province of Newfoundland and Labrador (NL), Canada, were currently in practice (i.e., involved in direct patient care), and regularly managed patients with LBP were eligible for this study. Both GPs and chiropractors routinely manage patients with LBP and can order imaging, particularly radiographs, within the province.

Purposive sampling was used to identify study participants. We chose this form of sampling to gather information from selected participants who could inform our understanding of strategies that could be used to enhance fidelity of training and delivery for the proposed intervention. Specifically, maximum variation was used to ensure the diverse views of participants were captured. Participants were recruited through professional and research networks and associations across NL using email. An emphasis was placed on seeking GPs and chiropractors from both urban and rural regions of NL and on seeking participants who may have differing views or practice patterns. At the end of each interview, participants were asked to identify an additional two people who may be interested in participating in the study (i.e., snowball sampling). Snowball sampling was used so that those who participated in our study would be able to identify other potential participants that they felt would be able to provide rich information.

Our sample size was informed by the principles for deciding saturation in theory-based interviews proposed by Francis et al. [29]. We conducted and analysed a minimum of 10 interviews to determine if we reached thematic saturation (i.e., the point where no new domains in the TDF were identified).

### Interview procedures

We conducted semi-structured interviews with open-ended questions with 10 participants (five GPs, five chiropractors). Five participants practised in an urban setting (three GPs, two chiropractors), while five participants practised in a rural setting (two GPs, three chiropractors). The participants were in practice for an average of 13 years (range 1–32 years). No participants refused to participate or dropped out of the study. Interviews were conducted by two members of the research team (DT and AP). One interviewer was a graduate student with limited experience in conducting interviews, and the other was a researcher trained in qualitative methods and interview techniques with over 15 years of experience. Both researchers have an interest in primary care and LBP research and one researcher (DT) is also a practising chiropractor. The experience of the primary investigator as a practising healthcare professional may have shaped the data collection (e.g., informing prompts used to participants’ responses), analysis (e.g., influencing deductive coding), and interpretation process (e.g., understanding the meaning of participant responses and relevant theoretical domains). Since participants were recruited through professional and research networks and associations that some of the research team members were members of, there was the possibility that some participants may have known the researchers prior to study commencement; however, participants only learned about the intentions and objectives of the interviews through the project information letter at the time of recruitment.

Interviews were conducted over a videoconferencing platform, Cisco Webex (Cisco Systems, Milpitas, United States), with participants either at home or in their clinics. Virtual data collection was a suitable method to reach participants from both rural and urban areas from across the province of NL. Reaching participants from diverse geographical locations was important because the practice needs and demands may vary across locations. The primary investigator has also worked with virtual care delivery platforms, bringing experience in communication through virtual formats. Interviews took between 50 to 65 min. No repeat interviews were carried out. The following demographic questions were collected at the start of the interview: profession (GP or chiropractor); practice location (urban or rural); and number of years in practice. The primary investigator (DT) then provided a brief presentation on intervention fidelity (what it is and why it is important), the aims of the interview, and proposed strategies to enhancing fidelity to provider training and delivery for the proposed intervention. All interviews were audio-recorded and transcribed verbatim by the primary investigator (DT). Approximately 9.5 h of recordings were transcribed for analysis. No additional researchers or observers were present during the interviews and field notes were not taken. Transcripts were not returned to participants for comments or correction. The collective views of the participants were taken into consideration during data analysis and interpretation, so member checking of their transcripts or the interpretation of their data would not be appropriate. Additionally, the overall aim of this study was to inform intervention developers on how to enhance intervention fidelity for the intervention that is being developed, meaning the research team’s interpretation of the findings may be more relevant.

### Interview guide

The interview guide (Additional file 2) was adapted from a previous study which aimed to develop an intervention fidelity protocol for an intervention to promote self-management for people with chronic LBP or osteoarthritis [26]. The NIHBCC intervention fidelity framework was also used to guide the develop of our interview guide, as it provided specific strategies which could be used to enhance fidelity to provider training and intervention delivery [15]. Participants were asked specifically about their thoughts on (including barriers and enablers) various strategies to enhance fidelity to provider training and intervention delivery for the proposed intervention. Credibility of the interview guide was established through multiple content experts in qualitative research (HE), intervention fidelity (ET), and LBP. The interview guide was pilot tested with two participants and refined to include additional prompts and probing questions.

### Data analysis

Data analysis was guided by the Theoretical Domains Framework (TDF) [27], which contains 14 theoretical domains, covering 84 theoretical constructs [30]. The TDF is a theoretical framework designed for the implementation of evidence-based practice [27] which has been used across health behaviour change research to identify influences of (i.e., barriers and enablers) specific health professional behaviours [31]. Data was analysed using a three-step process: [1] domain coding; [2] generating specific belief statements; and [3] identifying relevant domains [31]. Data collection and data analysis were completed iteratively; participant responses and belief statements generated by the researchers (described below) were used to guide the probing questions within the interview guide. The data was discussed at all stages by the research team and consensus on the coding, belief statements, and relevant domains was reached throughout the data analysis process.

#### Domain coding

The TDF was used as the coding framework to code and analyse the data following methods outline in the TDF guide [31]. Data was first analysed deductively, where interview transcripts were coded into the domains of the TDF. Prior to the start of coding, the primary investigator (DT) developed a codebook for each domain in the TDF (Additional file 3). The codebook was reviewed by another research team member experienced in coding interview data using the TDF (AMP). The codebook was also refined with the coding of additional interviews. Coding began after two interviews were conducted. Interviews were coded using NVivo (V12, QSR International, Melbourne, Australia). Two coders (DT and RL) independently read the transcripts until they were familiar with the data prior to beginning coding. The reviewers independently coded participant responses into one or more of the 14 relevant theoretical domain(s). To do this, the coders considered the content of the participant responses in relation to the definition of each theoretical domain. Only participant responses relating to the target behaviour of “being trained in and delivering an intervention to reduce imaging for LBP with high fidelity” were coded. The coders met for consensus after coding each interview and a third member of the research team (AP) was consulted if discrepancies persisted.

#### Generating specific belief statements

Data was then analysed inductively, with one coder (DT) generating statements representing the key message of each response (i.e., a specific belief). The list of specific beliefs was reviewed by another member of the research team (AP) for completeness and accuracy.

#### Identifying relevant domains

One coder (DT) identified the domains representing key barriers and enablers to enhancing fidelity to provider training or intervention delivery of the proposed intervention. The domains most likely representing perceived barriers and enablers were identified through considering the frequency of the belief statements, the presence of conflicting beliefs (i.e., participants reporting mixed views for a particular strategy to enhance fidelity to provider training or intervention delivery), and the perceived strength of the impact a belief may have on enhancing fidelity to provider training and intervention delivery (i.e., participants expressing beliefs they were particularly vocal about determined by length of participant quote or the use of emphatic or emotional speech) [31, 32]. Using these criteria, the research team decided to take a more conservative approach to considering domains as non-relevant. We determined that domains were non-relevant if no participant quotes were coded to that domain, or if only one participant expressed this belief and the perceived strength of this belief was low (identified by less text and if they did not demonstrate any emphatic or emotional speech). The relevant and non-relevant domains were checked by another member of the research team (AP).

## Results

### Barriers and enablers to enhancing fidelity of provider training

The proposed intervention involves asking GPs and chiropractors to use an educational booklet with a clinician-patient decision aid, reminders of indications for imaging for non-specific LBP, and suggestions for providing evidence-based, patient-specific self-management strategies. To ensure the GPs and chiropractors understand the intervention and feel confident in delivering it as intended, a training session is proposed before rolling out the intervention in community clinics. The training session, which we were interested in getting feedback on, includes strategies to enhance learning, such as role play, using a participant training manual, and potential booster sessions. Specific to the domain of intervention fidelity related to provider training, we aimed to understand the barriers and enablers to the behaviour of attending the training session, followed by the behaviour of participating in the different training session strategies. As such, the barriers and enablers to both behaviours are described separately.

#### Relevant domains

Our analysis revealed various barriers and enablers to attending training related to the following domains: [1] Beliefs about capabilities, [2] Optimism, [3] Reinforcement, [4] Memory, attention, and decision processes, [5] Environmental context and resources, [6] Emotion, and [7] Behavioural regulation. The specific beliefs with illustrative quotes for each of the relevant domains are presented in Table 1.

**Table 1:** Barriers and enablers (including belief statements and sample quotes) of fidelity to the proposed provider training for relevant domains

Domain	Belief statement (Enabler/Barrier)	Sample quotes	Frequency (out of 10)
Beliefs about capabilities	I am confident in my ability to use an algorithm and/or provide a resource to patients without training. (*Barrier*)	“What you’re proposing doesn’t really sound like it needs much training. Like I think a lot of physicians hopefully already know this and if they’re given an algorithm to follow, it sounds like it should be pretty straightforward.” GP004	2
Optimism	I think the proposed training strategies (including role play, a training manual, and booster sessions) will help to ensure I feel trained in using the intervention as intended. (*Enabler*)	What do you think about some of these potential strategies for ensuring fidelity related to training? “I think they’ll work.” GP001 “I think they’re awesome.” DC005	6
		“I think they’re all very reasonable…including the role play.” GP002	
		“I think that [a training manual] would be helpful. Every bit of training is helpful. It’s better that the practitioner has something to review and read before going into it blindly.” DC002	
		“I think booster sessions would be really good.” DC002	
	I think this intervention is great and would want to participate in training and delivering this intervention. (*Enabler*)	“And so I’m looking at this and I think this is phenomenal. Right? And you could follow and see what has changed in your practice and this is where I get all excited about quality improvement because I think it’s phenomenal.” GP001	4
		“Having this conversation about research, I’m like excited – like ‘Yes, I want to do this, that would be awesome’.” DC004	
Reinforcement	Incentives (e.g., continuing education credits, monetary compensation) would help me to attend training as intended. (*Enabler*)	“It has to be CME (continuing medical education) accredited. Absolutely. You might get a few people doing it if it’s not, but it’s gotta be CME accredited.” GP001	9
		“Well people love CE (continuing education) hours. If there’s any way to get a simple set up for CE to it, that’s always an incentive for people.” DC003	
		“Honestly, being compensated for time. Because if physicians have to take time away from their practice to do this, you know, you get paid to do work. So being compensated per hour that you spend in [training] would probably increase participation.” GP002	
Memory, attention, and decision processes	I will not participate in this training if I do not see the benefit to my clinical practice. (*Barrier*)	“At the end of the day, there has to be a direct benefit to us as family physicians as to do we…like we all want to provide good care. But either this helps me cut the conversation shorter or helps me get the patient on my side quicker, right? So something has to help me be better than what I’m currently doing.” GP003	2
		“I mean my gut is I don’t think I would personally sign up for any kind of training session because I already engage [in other continuing education]. I just don’t have enough time in my schedule to do that, especially where it’s so specific to one type of intervention…just considering the grand scope of what we see in a day.” GP005	
Environmental context and resources	Logistical issues (e.g., time, scheduling, location of training and associated expenses) would prevent me from attending training sessions. (*Barrier*)	[On challenges to attending training sessions] “It’s just finding time in your private practice. That’s it. Just time management.” DC002	9
		“I just don’t have enough time in my schedule to do that [training], especially where it’s like so specific to one type of intervention.” GP005	
		““It’s difficult to come in and do these sessions. It’s an expense. For me to go back and forth to the capital city, just in gas, if somebody had to stay in a hotel… So the challenge would be to get the rural people into the urban centre where you’re most likely to have these in-person sessions.” DC005	
Emotion	Provider burnout is a barrier to attending training for this intervention. (*Barrier*)	[On challenges to attending training sessions] “I think it’s provider burnout.” GP004	3
		[On attending training sessions] “It would be very daunting. I would feel overwhelmed by having to commit to extra training in order to use an intervention that’s supposed to either reduce time in my practice or make my quality of care better or improve the outcomes for my patients.” GP005	
	Family physicians may be offended by being asked to participate in training for an intervention to reduce LBP imaging. (*Barrier*)	“And I think community physicians then get a bit jaded and they kind of get their backs up like “what do you mean, I don’t know enough about LBP?!”. You know what I mean? It just becomes kind of one more thing that we’re being told that we’re not very good at and we need to get better, which is true – we’re not very good at this. I will be the first to admit it. But in a system that doesn’t support our community family physicians very well, it can come across as being critical and can be taken incorrectly by those physicians I think.” GP001	1
Behavioural regulation	The logistics of training (e.g., length of sessions, flexibility in scheduling, synchronous and asynchronous options) will help clinicians to complete the intervention training. (*Enabler*)	[On ways to overcome time or scheduling challenges] “Options. Being able to give a couple different options for people as to when they can attend. So they look at their calendar, these 3 options are out for me but I can make this one. Rather than it’s happening this day at this time. I think being able to give people a little bit of leeway to figure out what time works best.” DC003	8
		“You’re going to have to make it very efficient…and I think a little bit of an a la carte, where I can do what I feel like I need to do. … I think you just need a tailored…or maybe multiple sessions so like I can attend the training session, but not attend the role playing session because I feel like I don’t need that one in particular, right?” GP003	
		[On the ideal training time of a training session] “I would say no more than 2 h. All together, in one sitting. Any more than that, it’s kind of hard to get people to commit to. So I would say no more than 2 h. You can do it split up, like you could do two 2-h sessions if you felt like that much training was needed, but no more than 2 h at a time.” GP001	
		“I’ve seen the transportation from face to face to online/web courses. Anything people can do at say 9 pm when they’re home from work, online. I’m looking for convenience. If I can do it online, that’s perfect. This face-to-face stuff and traveling is done. A module, you review it and click – completed, completed, completed. And you can’t finish that section until you’ve read it and check down ‘yes I’ve done it’.” DC002	
	A training manual (e.g., with charts and visuals, digital version) to review and refer back to on my own time would help me to train for using this intervention. (*Enabler*)	“But I do like the possibility of having something like a manual that if it’s 8 o’clock on Monday night and I’m just sitting doing work, can I pull that up and just refresh that way? So having a manual but then also sitting in with everybody.” DC003	5
		“PDF. I’ll lose the paper. I never have the paper. I basically have, on my desktop computer, a folder for almost everything. Like I said, the moment it becomes not up to date then people will stop using it. So even if it’s a webpage that I could just link to and it allows people to keep it up to date. Because if it’s not up to date, people will just stop using it. A PDF is harder for you to keep distributing, while the web you can just update it.” GP003	
	The opportunity to meet in-person in a group setting would help me to train for this intervention. (*Enabler*)	“Well I’m Zoom’ed out. … So the reality is that if and when we can get together more, I think that a group setting in a real, in-person group setting, in a room, I think would be way more beneficial.” DC005	1

The relevance of a domain was determined through the consideration of the frequency of the belief statements, the presence of conflicting beliefs, and the perceived strength of the impact a belief may have on enhancing fidelity to provider training

GP: General Practitioner; DC: Doctor of Chiropractic; LBP: low back pain

#### Fidelity to provider training: behaviour of attending training

Barriers: Five barriers related to attending training for this type of intervention were identified by participants. The greatest barrier was related to logistical issues preventing participants from attending the training sessions (*Environmental context and resources*). For example, almost all participants believed that a lack of time, and a training session that was not flexible to their schedules, would be a challenge for them to attend. In-person training sessions were also thought to be a logistical challenge because they may be more difficult for clinicians working in rural areas of the province to attend if they were held in the capital city of the province. Some participants reported that they already felt confident in their ability to deliver this type of intervention, so they would not need to attend training (*Beliefs about capabilities*), and one participant suggested that family physicians might take being asked to train for an intervention to reduce a commonly encountered issue like LBP as being critical of their existing skillset and may feel offended (*Emotion*). Participants also felt that a barrier for attending training was clinician burnout, as they already had a lot of professional commitments and felt that attending training sessions would be daunting and overwhelming (*Emotion*). Participants also suggested that they would not participate in training for this type of intervention if they did not see it benefitting their clinical practice (e.g., if the intervention did not help shorten their conversation with patients about why imaging for LBP is not indicated) (*Memory, attention, and decision processes*).

Enablers: Three enablers related to attending training for this type of intervention were identified by participants. The greatest enabler was related to providing an incentive to attend training (*Reinforcement*). Continuing education credits was the most popular type of incentive discussed; other suggestions for incentives included monetary compensation for time away from work and offering catered events during the training sessions. Participants suggested some strategies that would help with overcoming logistical issues for training (*Behavioural regulation*). These strategies included having training sessions that were of a shorter duration and flexible session offerings that clinicians could choose from based on their schedules. Another suggested strategy was offering the training both synchronously and asynchronously, such as having a pre-recorded webinar or online course clinicians could complete on their own time followed by a live session with instructors to practise skills required to deliver the intervention. Participants generally felt optimistic about being trained in using this intervention, as they believed it to be a much-needed quality improvement initiative for their profession and were also excited to contribute to research (*Optimism*).

#### Fidelity to provider training: participation in suggested training session strategies (e.g., role play, using a participant training manual, and potential booster sessions)

Barriers: Participants reported that, once they could attend the training session, there were no perceived barriers to participating in the suggested training session, which may include strategies such as role play, a participant training manual, and/or booster sessions.

Enablers: Three enablers related to participating in the suggested training session strategies were identified by participants. Participants were generally optimistic that the proposed training session strategies (e.g., role play, participant training manual, potential booster sessions) would help them to feel trained in using the intervention (*Optimism*). Participants felt that having a manual they could review and refer to on their own time would help them to train in using the intervention (*Behavioural regulation*), and one participant felt that in-person training sessions would be more beneficial to them because they felt burned out from virtual training (*Behavioural regulation*).

#### Non-relevant domains

Our analysis revealed that barriers and enablers related to the domains of [1] Knowledge, [2] Skills, [3] Social, professional role and identity, [4] Beliefs about consequences, [5] Intention, [6] Goals, and [7] Social influences were not relevant to enhancing fidelity to provider training of the proposed intervention. No data were coded at the domains of Knowledge, Skills, and Intention. One participant felt that the training for this intervention could be a quality improvement initiative, which they considered an important part of their profession as a family physician (*Social, professional role and identity*). One participant felt that virtual training would be challenging to participate and engage in (*Beliefs about consequences*). One participant believed that an in-person training session was important to ensure that clinicians were invested in the intervention (*Goals*) and that they would benefit from participating in group training sessions with other colleagues (*Social influences*). The specific beliefs with illustrative quotes for each of the non-relevant domains are presented in Table 2.

**Table 2:** Barriers and enablers (including belief statements and sample quotes) of fidelity to the proposed provider training for non-relevant domains

Domain	Belief statement (Enabler/Barrier)	Sample quotes	Frequency (out of 10)
Knowledge	No relevant quotes coded to this domain		
Skills	No relevant quotes coded to this domain		
Social, professional role and identity	Quality improvement is an important part of my profession, as a family physician. (*Enabler*)	“This [training] is exactly what we want to be doing and this is quality improvement.” GP001	1
Beliefs about consequences	I think virtual training would be challenging to participate in and really engage in. (*Barrier*)	“I think the challenge is right now, certainly, with Zoom. I think the challenge is that people are Zoom’ed out, just like me. They just don’t want to be at it anymore. And I think that’s going to be a huge challenge if you choose to do it like that. Because half of communication is body language, nuances, facial features, you know. Communication on Zoom is very difficult and you lose a big chunk of it. And if you’re doing a group session, you’re reading body language, you’re reading little nuances in the way they look or giggle or whatever. And that’s feedback – you know, positive or negative, it’s still feedback. So that challenge I think is going to be there with Zoom.” DC005	1
Intention	No relevant quotes coded to this domain		
Goals	An in-person training session is important to ensure clinicians are invested in the intervention. (*Enabler*)	“But I’m a sucker for having everybody on the same page, so to have a session and know that everybody’s there and everybody’s kind of paying attention and really invested in it. I always like that a little bit more.” DC003	1
Social influences	It would benefit me to participate in group training sessions with other colleagues. (*Enabler*)	“It is actually nice to be able to sit down with your colleagues and go over this kind of stuff and hear different situations that other people have been in, similar to what you are.” DC003	1

The relevance of a domain was determined through the consideration of the frequency of the belief statements, the presence of conflicting beliefs, and the perceived strength of the impact a belief may have on enhancing fidelity to provider training

GP: General Practitioner; DC: Doctor of Chiropractic

### Barriers and enablers to enhancing fidelity of delivery

As previously described, the proposed intervention involves asking GPs and chiropractors to use a clinical resource consisting of an educational booklet with a clinician-patient decision aid/algorithm, reminders to indications for imaging for non-specific LBP, and suggestions on providing evidence-based, patient-specific self-management strategies. Specific to the domain of intervention fidelity related to intervention delivery, we aimed to understand the barriers and enablers to the behaviour of delivering the intervention by GPs and chiropractors to their patients.

#### Relevant domains

Our analysis revealed various barriers and enablers related to the following domains: [1] Beliefs about capabilities, [2] Optimism, [3] Goals, [4] Memory, attention, and decision processes, [5] Environmental context and resources, [6] Social influences, and [7] Behavioural regulation. The specific beliefs with illustrative quotes for each of the relevant domains are presented in Table 3.

**Table 3:** Barriers and enablers (including belief statements and sample quotes) of fidelity to the proposed intervention delivery for relevant domains

Domain	Belief statement (Enabler/Barrier)	Sample quote	Frequency (out of 10)
Beliefs about capabilities	I am confident I can deliver this intervention as planned. (*Enabler*)	How easy or difficult do you think it would be to adhere to delivering say 80% of the intervention components? “I think it would be pretty easy to do that, yeah.” DC004	6
		“I don’t think 80% is too hard.” GP003	
	I would not be confident in delivering this intervention as planned in certain situations (e.g., pushback from patients, if I had to educate on self-management strategies, if I was limited on time, if patient had other reasons for presenting for care). (*Barrier*)	“As I had alluded to earlier, my patients rarely ever book an appointment just to talk about their back pain. And so what inevitably would happen, even if we did just book an appointment for their back pain, is that we would get partway through this, and it would make them think about…something else…and then we would end up totally off topic. And so it can be difficult in going through an intervention like this to try to keep it contained, because it’s difficult to keep anything contained, and so that can be tricky.” GP001	5
		“I mean…self-management strategies are always a difficult conversation…difficult to deliver to a patient. And reassurance and education. I mean, they’re so important but they are the things that take the longest I find in practice. And often times, patients aren’t always willing to do the self-management techniques at home” DC004	
	I am confident I can deliver this intervention during my clinic encounter, without being worried about time. (*Enabler*)	“I can manage to get a lot of the information out in a reasonable amount of time.” GP002	1
Optimism	I believe the proposed intervention delivery enhancement strategies (e.g., algorithm, use of a script) would help me to deliver the intervention. (*Enabler*)	[On the strategies for enhancing fidelity to intervention delivery] “I like all of them.” DC003	4
		“I think it’s great. I love algorithms.” GP004	
		“I like the script idea.” DC003	
		“[Self-management strategies] is the part I look most forward to. That’s the part I want the most out of. Because like I said, patients don’t get an x-ray but then they leave with some really good information of things that they can do to help with their cause. So that’s the thing that would get you the buy in for the whole program.” GP003	
Goals	It is important for me to deliver the intervention as planned. (*Enabler*)	[On the importance of delivering the intervention as planned] “It’s very important. I think it’s very necessary.” DC002	7
	Delivering this intervention as intended is only important to me if I believe non-indicated imaging is an important issue and if the intervention aligns with the appropriate standard of care I provide. (*Barrier*)	“How big of a problem a particular individual provider views imaging for lower back pain…how important they think it is to their practice is going to decide whether or not they use the tool or are committed to it.” GP001	2
		“I think that my priority is always am I giving the standard of care that’s appropriate to the clinical situation to my patient. So if this situation looked like it would be appropriate to fit the intervention, then I would use the intervention to the best that I could to meet the clinical scenario.” GP005	
Memory, attention, and decision processes	Features of the training for this intervention (e.g., in-person training session, use of role play, training manual, booster sessions) would help me remember how to deliver the intervention as intended. (*Enabler*)	“But I’m a sucker for having everybody on the same page, so to have a session and know that everybody’s there and everybody’s kind of paying attention and really invested in it.” DC003	6
		“I mean, there’s no question role playing is very important. I mean, you could read something and it just quickly dissipates from your brain as time goes. And if you solidify that with a concrete learning example like role playing, I think it’s essential.” DC005	
		[On the importance of a training manual] “Then if I do this once every 3 months or once every 9 months, I don’t have to try to remember what I did at the last session, but I could quickly go in 2 min and review things myself, and then it’s very fresh when I see the patient. So to me, for someone who’s busy, that would be super super helpful.” GP004	
		“But definitely, the regular booster sessions as well help if it’s a study that’s going over a long period of time. People sort of lose and forget what they’re doing and sometimes just that meeting to make sure people are still on the right track is good.” GP002	
	Proposed features of the intervention (e.g., algorithm, script for delivery or patient discussions, session checklist) and reminders of the intervention components built into the electronic medical record would help me remember how to deliver the intervention as intended. (*Enabler*)	“The clinical resource is a definite huge bonus. Anything you can reach to quickly give yourself a refresher or make sure you’ve checked all your bases is nice.” DC003	4
		“Having a checklist as part of the resource, that these are your main talking points and I’ve got it printed out or pinned up in the office that if the conversation comes up, I can look it over” DC003	
		“But I think just having a little bit of detail on the EMR, it would probably make sure that people remember it [the components of the intervention]” GP004	
		“The script for delivery is actually not bad because after a time, it becomes part of your normal lingo, right? So you start off sort of mechanically, I guess, in a way. Sort of saying ‘this is what we’re doing blah blah blah’. It eventually becomes part of what your dialogue is.” GP002	
	I may not deliver the intervention as intended because I already have or will develop my own way of explaining concepts around imaging and LBP. (*Barrier*)	“I think too, once I’ve implemented it, like for example, once I’ve used the tool, let’s say we use the clinician decision tool. Once I’ve used it once or twice, I don’t need to bring it up every time, because I’ve got it right? So like, the main part would be like, I guess…because it’s very much dependent on the patient. So like there’s some flexibility in how that program is also delivered. So like, did I use the decision making tool today? Well I didn’t take out the decision making tool and look at it, but I did use it in my head.” GP003	3
		“I think similar to before, just having this become your autopilot vs. what I use right now when this conversation comes up. It’s remembering to switch to this, which I guess in reality, is not too far different from what I already do, but for some people, maybe it would be a bit different.” DC003	
	I may not deliver the intervention as intended if it takes too much of my time. (*Barrier*)	“But the moment it becomes cumbersome or takes more time, because time is ultimately the factor that not a lot of us have a lot of, and the moment that it becomes more time to do it, it will become less utilised properly.” GP003	2
Environmental context and resources	Lack of time may be a barrier to delivering the intervention as planned. (*Barrier*)	“The biggest problem when it comes to clinical resources or decision tools or whatnot in family medicine is that we don’t have any time.” GP001	6
		“Time would definitely be a challenge. As a chiropractor, I know most only spend about 20 min with their patient and that’s for a quick re-assessment, a conversation, and treatment.” DC004	
	Time is not a barrier to delivering the intervention as planned. (*Enabler*)	“I’m not in fee-for-service anymore so I have the time to explain things well.” GP004	1
Social influences	Patient pressure to order imaging will not prevent me from delivering this intervention. (*Enabler*)	[On patients being persistent on getting an x-ray] “There’s some, but usually, when you talk about it, they come around to it.” GP002	5
		“I think that would be something to contend with, but I don’t think it would prevent me from [delivering the intervention]” DC001	
	Patient pressure for imaging may influence my ability to deliver this intervention as intended. (*Barrier*)	“Because ultimately, I find, it’s not my clinical decision tools to know whether or not to do an x-ray that’s the issue. My issue is the patient demanding to have an x-ray. That is ultimately what it brings it down to. … sometimes it’s easier to not fight the fight and just say ‘Here’s your x-ray because you’re not going to leave until you get one anyways’.” GP003	3
		“But you’ll always hit difficult people who want what they want regardless of whether it’s going to be the most effective resource for them. So I think the biggest challenge you’ll get is just personality or patient types.” DC003	
Behavioural regulation	An intervention script with key talking points (that isn't too prescriptive) would help me to deliver the intervention as intended. (*Enabler*)	“Every practitioner has their own style of delivery. Just key points that have to be delivered. I think that would be the best way to do it.” DC002	10
		“Every person is their own illness experience. Not everybody experiences low back pain the same way. … And I agree there’s some people who would find that this is difficult to understand, so that may make it a bit of a challenge to delivering this – patients themselves. So you need to have a little bit of flexibility in the script and how you’re delivering it.” GP002	
		“I think speaking points because you would make your own way in how to do it. But you want to be able to touch on all the main things that you want to get into the session.” GP003	
	I think having regular check-in times (e.g., booster sessions, progress check-in emails) and/or the ability to reach out to the research team (e.g., clinical coach or project champion) when needed would help me to deliver this intervention as intended. (*Enabler*)	“I really like the idea of a booster session. It makes a lot of sense. There are so many CME events that I go to and then I get all excited and I take it away and then I go to implement it into my practice and then it kind of falls apart… And a booster session that includes a component of bringing back difficult encounters or you know… ‘I tried to use this tool on this patient and here’s what happened. How could I approach that better next time or what did I do wrong?’. I think that would be very useful. … I think you have 1 booster session maybe 6–8 weeks out and that would be the best that we would be able to hope for when it comes to buy in and engaging people right now.” GP001	10
		“I think having a champion is a really good idea … if you had somebody that basically said ‘I’m trained up on this. I’m happy and interested in helping’.” GP001	
		“Having that touch base call every 4–6 weeks sometimes does get people get back on track and make them think about what the purpose of this is and the flow and answer any questions that they may have.” GP002	
		“I don’t know if I would spend time putting on a booster session. I would more say, ‘This is our contact information. If you have an issue, then reach out to us.’.” GP003	
		[On receiving support from the research team] “Regular emails, just reaching out to see if they need any assistance, see how their progression is.” DC002	
	Tailoring the intervention to fit within a regular appointment time (5–10 min for GPs; 15–20 min for DCs) will help me to deliver the intervention as intended. (*Enabler*)	“If [LBP] were the only thing in the appointment, it would be ideal. Because in that case, then you could go through the whole thing about the indications and the education and the thing goes along with it, and then running through some of the interventions that they can do themselves to get started.” GP002	5
		“I think if the script could be honed enough that it could be all done in 15–20 min for us anyways. It would be about fitting it into an appointment time.” DC001	
	Flexibility in intervention material formats (e.g., access to both digital and paper copies with the possibility of having digital copies built into the electronic medical record) would help me to use the materials as intended. (*Enabler*)	“What I’m picturing is in the EMR or on a website, you can just bring up the tool and print it right from the computer. That way if you’re moving around multiple clinic rooms and things, you don’t have to have multiple booklets and they get lost and stuff like that.” GP001	3
		“I’d say most people are pretty tech savvy at this point. So I’ve had good success with links and stuff, or just recommending go on YouTube and search this. Just the ease of being able to send that off. But a hard copy is…it’s easy to give out and there’s no barrier at that point. For anybody who doesn’t have access to Internet or just doesn’t go on as much to it. It eliminates all barriers.” DC003	

The relevance of a domain was determined through the consideration of the frequency of the belief statements, the presence of conflicting beliefs, and the perceived strength of the impact a belief may have on enhancing fidelity to provider training

GP: General Practitioner; DC: Doctor of Chiropractic; EMR: electronic medical record; CME: continuing medical education; LBP: low back pain

Barriers: Some participants reported that they would not be confident in delivering the intervention as planned in certain situations (e.g., if they were short on time, received pushback from patients, if they had to educate on self-management strategies) (*Beliefs about capabilities*). Some participants believed that since they already had their own ways (or would develop their own ways of explaining why imaging is not indicated to patients), they may not stick to a particular script and thus may not deliver the intervention as intended (*Memory, attention, and decision processes*). Some of the GPs in our sample also reported that delivering the intervention as intended was only important if they believed non-indicated imaging was an important issue and if they thought the intervention aligned with the appropriate standard of care they already provided for patients with LBP (*Goals*). Most participants believed a lack of time would be a barrier for delivering the intervention as planned (*Environmental context and resources*), with some participants reporting that they would not deliver the intervention as intended if it took too much time (*Memory, attention, and decision processes*); however, one participant did not feel that time would be a barrier to delivering the intervention in their practice (*Environmental context and resources*) and another participant felt confident in being able to deliver the intervention as planned, without being worried about time (*Beliefs about capabilities*). Lastly, some participants identified that patient pressure/demands for imaging would influence their ability to deliver the intervention as intended (*Social influences*), although other participants did not believe that patient pressure would influence their ability to deliver the intervention as intended (*Social influences*).

Enablers: Overall, participants felt the proposed intervention delivery enhancement strategies (e.g., clinical algorithm, script) were great ideas and would help them to deliver the intervention (*Optimism*). They were also confident they could deliver the proposed components as planned (*Beliefs about capabilities*). Participants reported that delivering the intervention as planned was important to them, with many understanding that doing otherwise compromises the study and any value that can be gained from implementing the intervention (*Goals*).

Many participants felt that features of the training for this intervention (e.g., having a training session, using role play, having a participant training manual, having booster sessions) would help them to remember how to deliver the intervention as intended (*Memory, attention, and decision processes*). For example, having a participant training manual that they could refer to would allow them to quickly review the content before delivering the intervention. Additionally, all participants suggested that having regular check-in times would help them to deliver the intervention as intended. However, the mode of check-in varied from group-based booster sessions to progress emails from the research team to having the ability to reach out to the research team via a clinical coach or champion when needed (*Behavioural regulation*).

Participants also felt that the proposed features of the intervention itself (e.g., algorithm, script for patient discussions, session checklist) and reminders of the intervention components potentially built into the electronic medical record would help them to remember how to deliver the intervention (*Memory, attention, and decision processes*). All participants suggested that having a script with key talking points (instead of a word-for-word script) that would allow for flexibility in how they discuss with their patients would help them to deliver the intervention as intended (*Behavioural regulation*). Some participants also suggested that having some flexibility in the intervention material formats would help them to actually use the intervention material as intended, with some preferring digital copies, others preferring paper copies, and others preferring digital copies built into the electronic medical record (*Behavioural regulation*). Participants in our study also suggested that tailoring the intervention to fit within a regular appointment time (e.g., 5–10 min for GPs and 15–20 min for chiropractors) would enable them to deliver the intervention as intended (*Behavioural regulation*).

#### Non-relevant domains

Our analysis revealed that barriers and enablers related to the domains of [1] Knowledge, [2] Skills, [3] Social, professional role and identity, [4] Beliefs about consequences, [5] reinforcement, [6] intention, and [7] emotion were not relevant to enhancing fidelity to provider training and delivery of the proposed intervention. No data were coded at the domains of knowledge, skills, social, professional role and identity, beliefs about consequences, and intention. One participant believed that the established clinical routines of clinicians may make delivering the intervention as intended more difficult, explaining that breaking those clinical habits to implement new changes would be a difficult process (*Reinforcement*). One participant felt they would feel comforted by having a training manual they could reference to deliver the intervention as intended (*Emotion*). The specific beliefs with illustrative quotes for each of the non-relevant domains are presented in Table 4.

**Table 4:** Barriers and enablers (including belief statements and sample quotes) of fidelity to the proposed intervention delivery for non-relevant domains

Domain	Belief statement (Enabler/Barrier)	Sample quote	Frequency (out of 10)
Knowledge	No relevant quotes coded to this domain		
Skills	No relevant quotes coded to this domain		
Social, professional role and identity	No relevant quotes coded to this domain		
Beliefs about consequences	No relevant quotes coded to this domain		
Reinforcement	Clinicians' established practice routines may make delivering the intervention as intended challenging. (*Barrier*)	“I think similar to before, just having this become your autopilot vs. what I use right now when this conversation comes up. It’s remembering to switch to this, which I guess in reality, is not too far different from what I already do, but for some people, maybe it would be a bit different.” DC003	1
Intention	No relevant quotes coded to this domain		
Emotion	I would feel comforted by having a training manual to refer back to in order to know that I am delivering the intervention as intended. (*Enabler*)	[On the importance of a training manual] “For me, I think it would provide more comfort. I think instead of being something seen as a time consumer, I think I’d feel that as least I was being thorough and that I wasn’t missing anything.” DC001	1

The relevance of a domain was determined through the consideration of the frequency of the belief statements, the presence of conflicting beliefs, and the perceived strength of the impact a belief may have on enhancing fidelity to provider training

GP: General Practitioner; DC: Doctor of Chiropractic

## Discussion

We conducted a qualitative study which interviewed 10 GPs and chiropractors on their perceived barriers and enablers to enhancing fidelity of training and fidelity of delivery for an intervention aimed at reducing non-indicated imaging for LBP. Data analysis was guided by the TDF, a determinant framework in implementation science commonly used to examine factors influencing implementation. Barriers and enablers to enhancing fidelity to provider training were related to seven domains in the TDF, with a variety of barriers and enablers described by participants. The main barriers for attending training centred around a lack of time to attend and some participants feeling they did not need to attend either because they already felt confident in managing patients with LBP without imaging or because they did not see the benefit to using this type of intervention in their clinical practice. The main enablers for attending training were having incentives to attend and having flexibility in the training scheduling and format. Barriers and enablers to enhancing fidelity to delivery related to seven domains in the TDF, again, with a variety of barriers and enablers described by participants. A barrier was that participants may not deliver the intervention as intended because they had established habits on how to discuss why imaging for LBP was not indicated; however, some enablers suggested by participants included having a flexible script with key talking points and regular check-ins with the research team to ensure they were delivering the intervention as intended. Time and patient pressure were believed by most to be barriers to delivering the intervention as intended, and participants suggested that ensuring the intervention fit within the timeframe of their regular clinic appointment would help enable them to deliver the intervention as intended. Lastly, an enabler was that most participants recognised the importance of intervention fidelity and delivering the intervention as planned.

### Findings in context with existing literature

Few studies have explored the perceived barriers and enablers to enhancing provider training and intervention delivery for interventions aimed at reducing non-indicated imaging for LBP. The proposed intervention described in our current study is based on the intervention developed by Jenkins et al*.* [19, 33], which involves GPs delivering an LBP management booklet to patients to reduce non-indicated imaging for LBP. For that intervention, two studies exploring barriers and facilitators to implementation of the intervention were conducted, once during the intervention development process [19] and once during a feasibility study after GPs used the intervention with their patients [33]. A barrier identified in both studies was that delivering the intervention was time-consuming [19, 33], which was also identified as a perceived barrier for intervention delivery by most participants in our study. An enabler identified by both studies by Jenkins et al. was that GPs preferred both digital and paper formats for the intervention materials, as they believed digital formats were easier to store, could be kept up to date, and would serve as a reminder for them to use the intervention [19, 33], which were also perceived enablers in our study. While Jenkins et al. [33] did not explore barriers and enablers to attending training for the intervention, all GPs attended a training session, which was a 20-min individualised face-to-face session with a member of the research team. During the training session, GPs were provided with education on the appropriate use of imaging for LBP, an introduction to the LBP management booklet, and received a demonstration on how to use the booklet [33].

The perceived enablers for attending training that we identified in our study are similar to those identified in other studies on complex behaviour change interventions. Incentives are commonly used as an implementation strategy to improve practice behaviours of physicians [34], as well as within clinical trials to improve recruitment and retention of health professionals [35]; however, incentives may take a variety of formats, including continuing education, financial, or co-authorship. Additionally, in a study which used online training to train physical therapists in delivering an online, group-based program to patients with LBP, participants felt that virtual training sessions allowed for greater flexibility in scheduling [36]. However, they also felt that peer support and practice-based learning activities from face-to-face interactions were lacking [36]. These beliefs were also held by participants in our study, who believed there would be value in having both virtual and in-person training sessions as options.

All participants in our study believed that some form of check-in with the research team would be important throughout the period they were delivering the intervention (e.g., during a trial), although the methods they suggested for regular check-ins varied. Similarly, in the development of a fidelity protocol for a complex self-management intervention delivered by physical therapists, Toomey et al*.* [26] found that participants reported regular contact with the research team to prevent skill drift was acceptable. This resulted in including regular communication methods between the research team and physical therapists when the fidelity protocol was developed [26].

### Strengths

This was the first study to use the TDF to explore perceived barriers and enablers to provider training and intervention delivery for an intervention aimed at reducing non-indicated imaging for LBP. Using the TDF as our coding framework may allow for a theoretical explanation for the participants’ behaviours related to fidelity to provider training and intervention delivery.

Our sample included GPs and chiropractors from across the province of NL and found similar barriers and enablers. This might suggest that an intervention developed to reduce non-indicated imaging for LBP may be used by a range of health professionals. A number of strategies were used throughout data analysis to ensure the credibility and trustworthiness of the study findings. First, we have provided rich, detailed descriptions of each code/theoretical domain, as well as many supporting quotes. This should enable other researchers to judge whether findings are transferable to other behavioural domains and similar healthcare professional populations. To help ensure confirmability and dependability of our study findings, we retained a detailed audit trail of all data analytic decisions, as well as had regular team debriefing sessions on data decisions. Broadly, studies using an analysis based on the TDF follow a prescribed method of analysis, which was adhered to in our study; the detailed description of the data analysis methods used should also help other researchers replicate a study using the TDF in other health domains.

### Limitations

Since participants volunteered for the study, they may have been more likely to feel that non-indicated imaging was an important issue, potentially resulting in premature saturation. To avoid this, we specifically tried to target participants in different geographical regions of NL and when using snowball sampling, we specifically asked for additional participants with differing views. The interview guide was developed based on the NIHBCC fidelity framework, as we wanted to prioritise capturing key concepts related to intervention fidelity. However, the TDF was used as the coding framework for analysis, which may have resulted in less questions and responses directed at specific domains in the TDF. This may be a reason why some domains had no relevant participant quotes; future studies using the TDF for the primary analysis may consider using a TDF-based interview guide, as suggested by the TDF Guide [31]. The primary interviewer was not as experienced with conducting interviews and may not have asked enough probing questions, which may also have resulted in fewer relevant participant quotes at some domains. Additional pilot testing of the interview guide may have been needed to determine if more probing questions were needed.

### Implications for research and future directions

Our findings can contribute to the development of an intervention aimed at reducing non-indicated imaging for LBP by providing suggestions on how to enhance fidelity to provider training and intervention delivery. The strongest barriers related to attending training and delivering the intervention should be addressed. The training for this intervention should be flexible in its format and scheduling to accommodate for participants’ varied schedules, previous education and experience, and learning styles. An incentive would also need to be provided for participants to attend training. The delivery of the intervention should fit within a regular clinical appointment time (i.e., less than 15 min) and a variety of formats for delivery could be considered, including both paper and digital versions of the intervention. Various forms of reminders (e.g., reference to a participant training manual and flexible intervention script) should also be provided to participants delivering the intervention so they can more easily remember how to deliver the intervention and remember what the components of the intervention are. Participants would also likely benefit from follow up from the research team during the intervention delivery period in the form of contacting the research team on an as-needed basis. Our study revealed conflicting beliefs on patient pressure as a barrier to delivering the intervention as intended, which could be further explored in future research.

Our study highlights that it is feasible to conduct interviews with participants during the intervention planning phase to determine how intervention fidelity can be enhanced in a main intervention effectiveness trial. Future intervention trials should consider using this approach, as well as other implementation science methods, in the early stages of intervention development. This may impact the implementation and effectiveness of the intervention. The TDF is a useful implementation science framework that can be used to understand factors (i.e., barriers and enablers) that influence implementation outcomes, including intervention fidelity.

## Conclusion

We conducted a qualitative study with the overall aim of informing the design of a proposed intervention to reduce non-indicated imaging for LBP in Newfoundland and Labrador, Canada. Our first study objective was to explore barriers and enablers which were perceived to influence fidelity of training of GPs and chiropractors. Barriers and enablers to fidelity of provider training were related to seven TDF domains, with time as the largest barrier related to attending training and incentives and flexibility in the required training as the largest enablers. Our second study objective was to explore barriers and enablers which were perceived to influence fidelity of delivery of the proposed intervention. Barriers and enablers to fidelity of intervention delivery were related to seven TDF domains, with patient pressure, time, and existing habits as the main barriers related to being able to deliver the intervention as intended. Participants suggested various enhancement strategies that would improve their ability to deliver the intervention as intended, including having reminders on how to use the intervention and regular check-ins with the researchers. Our results may aid in the development of a more feasible and pragmatic intervention to reduce non-indicated imaging for GPs and chiropractors in NL. Exploring factors affecting intervention fidelity and ways to enhance intervention fidelity during the early stages of intervention development can help improve the results and interpretation of the main effectiveness trial for the intervention.

## Supplementary Information

**Additional file 1:** COnsolidated criteria for Reporting Qualitative research (COREQ) checklist.

**Additional file 2:** Interview guide mapped to the NIHBCC intervention fidelity checklist.

**Additional file 3:** Codebook for each domain in the Theoretical Domains Framework.

## Data Availability

The datasets used and/or analysed during the current study are available from the corresponding author on reasonable request.

## References

[1] Hoy D, Bain C, Williams G, March L, Brooks P, Blyth F (2012). A systematic review of the global prevalence of low back pain. Arthritis Rheum.

[2] Maher C, Underwood M, Buchbinder R (2017). Non-specific low back pain. Lancet.

[3] Chiarotto A, Koes BW (2022). Nonspecific low back pain. N Engl J Med.

[4] Hartvigsen J, Hancock MJ, Kongsted A, Louw Q, Ferreira ML, Genevay S (2018). What low back pain is and why we need to pay attention. Lancet.

[5] Henschke N, Maher CG, Refshauge KM, Herbert RD, Cumming RG, Bleasel J (2009). Prevalence of and screening for serious spinal pathology in patients presenting to primary care settings with acute low back pain. Arthritis Rheum.

[6] Oliveira CB, Maher CG, Pinto RZ, Traeger AC, Lin CWC, Chenot JF (2018). Clinical practice guidelines for the management of non-specific low back pain in primary care: an updated overview. Eur Spine J.

[7] Jenkins HJ, Downie AS, Maher CG, Moloney NA, Magnussen JS, Hancock MJ (2018). Imaging for low back pain: is clinical use consistent with guidelines? A systematic review and meta-analysis. Spine J.

[8] Kamper SJ, Logan G, Copsey B, Thompson J, Machado GC, Abdel-Shaheed C (2020). What is usual care for low back pain? A systematic review of health care provided to patients with low back pain in family practice and emergency departments. Pain (Amsterdam).

[9] French SD, Green S, Buchbinder R, Barnes H (2010). Interventions for improving the appropriate use of imaging in people with musculoskeletal conditions. Cochrane Database Syst Rev.

[10] Jenkins HJ, Hancock MJ, French SD, Maher CG, Engel RM, Magnussen JS (2015). Effectiveness of interventions designed to reduce the use of imaging for low-back pain: a systematic review. Can Med Assoc J (CMAJ).

[11] Lichstein KL, Riedel BW, Grieve R (1994). Fair tests of clinical trials: a treatment implementation model. Adv Behav Res Ther.

[12] Proctor EK, Powell BJ, McMillen JC (2013). Implementation strategies: recommendations for specifying and reporting. Implement Sci.

[13] Borrelli B, Sepinwall D, Ernst D, Bellg AJ, Czajkowski S, Breger R (2005). A new tool to assess treatment fidelity and evaluation of treatment fidelity across 10 years of health behavior research. J Consult Clin Psychol.

[14] Bellg AJ, Borrelli B, Resnick B, Hecht J, Minicucci DS, Ory M (2004). Enhancing treatment fidelity in health behavior change studies: best practices and recommendations from the NIH Behavior Change Consortium. Health Psychol.

[15] Borrelli B (2011). The assessment, monitoring, and enhancement of treatment fidelity in public health clinical trials. J Public Health Dent.

[16] Resnick B, Bellg AJ, Borrelli B, Defrancesco C, Breger R, Hecht J (2005). Examples of implementation and evaluation of treatment fidelity in the BCC studies: where we are and where we need to go. Ann Behav Med.

[17] Toomey E, Matvienko-Sikar K, Heary C, Delaney L, Queally M, Hayes CB (2019). Intervention fidelity within trials of infant feeding behavioral interventions to prevent childhood obesity: a systematic review. Ann Behav Med.

[18] Gorman G, Toomey E, Flannery C, Redsell S, Hayes C, Huizink A (2021). Fidelity of interventions to reduce or prevent stress and/or anxiety from pregnancy up to two years postpartum: a systematic review. Matern Child Health J.

[19] Jenkins HJ, Moloney NA, French SD, Maher CG, Dear BF, Magnussen JS (2018). Using behaviour change theory and preliminary testing to develop an implementation intervention to reduce imaging for low back pain. BMC Health Serv Res.

[20] Radiation Health and Safety Regulations under the Radiation Health and Safety Act. Consolidated Newfoundland and Labrador Regulation 1154/96 St. John’s, NL 2006. Available from: https://www.assembly.nl.ca/legislation/sr/regulations/rc961154.htm.

[21] Logan GS, Dawe RE, Aubrey-Bassler K, Coombs D, Parfrey P, Maher C (2020). Are general practitioners referring patients with low back pain for CTs appropriately according to the guidelines: a retrospective review of 3609 medical records in Newfoundland using routinely collected data. BMC Fam Pract.

[22] De Carvalho D, Bussières A, French SD, Wade D, Brake-Patten D, O'Keefe L (2021). Knowledge of and adherence to radiographic guidelines for low back pain: a survey of chiropractors in Newfoundland and Labrador, Canada. Chiropr Man Ther.

[23] To D, Hall A, Bussières A, French SD, Lawrence R, Pike A (2022). Exploring factors influencing chiropractors' adherence to radiographic guidelines for low back pain using the Theoretical Domains Framework. Chiropr Man Ther.

[24] Liu C, Desai S, Krebs LD, Kirkland SW, Keto-Lambert D, Rowe BH (2018). Effectiveness of interventions to decrease image ordering for low back pain presentations in the emergency department: a systematic review. Acad Emerg Med.

[25] Breitenstein S, Robbins L, Cowell JM (2012). Attention to fidelity: why is it important. J Sch Nurs.

[26] Toomey E, Matthews J, Guerin S, Hurley DA (2016). Development of a feasible implementation Fidelity protocol within a complex physical therapy–led self-management intervention. Phys Ther.

[27] Michie S, Johnston M, Abraham C, Lawton R, Parker D, Walker A (2005). Making psychological theory useful for implementing evidence based practice: a consensus approach. Qual Saf Health Care.

[28] To D, De Carvalho D, Pike A, Etchegary H, Patey A, Toomey E (2021). Exploring perceived barriers and enablers to fidelity of training and delivery of an intervention to reduce imaging for low back pain: a qualitative interview study protocol. HRB Open Res.

[29] Francis JJ, Johnston M, Robertson C, Glidewell L, Entwistle V, Eccles MP (2010). What is an adequate sample size? Operationalising data saturation for theory-based interview studies. Psychol Health.

[30] Cane J, O'Connor D, Michie S (2012). Validation of the theoretical domains framework for use in behaviour change and implementation research. Implement Sci.

[31] Atkins L, Francis J, Islam R, O'Connor D, Patey A, Ivers N (2017). A guide to using the Theoretical Domains Framework of behaviour change to investigate implementation problems. Implement Sci IS.

[32] Pike A, Patey A, Lawrence R, Aubrey-Bassler K, Grimshaw J, Mortazhejri S (2022). Barriers to following imaging guidelines for the treatment and management of patients with low-back pain in primary care: a qualitative assessment guided by the Theoretical Domains Framework. BMC Prim Care.

[33] Jenkins HJ, Moloney NA, French SD, Maher CG, Dear BF, Magnussen JS (2022). General practitioner experiences using a low back pain management booklet aiming to decrease non-indicated imaging for low back pain. Implement Sci Commun.

[34] Mostofian F, Ruban C, Simunovic N, Bhandari M (2015). Changing physician behavior: What works?. Am J Manag Care.

[35] Bower P, Brueton V, Gamble C, Treweek S, Smith CT, Young B (2014). Interventions to improve recruitment and retention in clinical trials: a survey and workshop to assess current practice and future priorities. Trials.

[36] Christou B, Sellars J, Barker K (2019). What are the experiences of therapists using the online Back Skills Training and implementing it within clinical practice?. Musculoskelet. Care.

