# Supplementary material for: A qualitative study exploring perceived barriers and enablers to fidelity of training and delivery for an intervention to reduce non-indicated imaging for low back pain
Source: Chiropr Man Therap. 2023 Jan 31;31:6. doi: 10.1186/s12998-023-00480-6 (PMC9890790; doi:10.1186/s12998-023-00480-6)
Supplement: Supplementary file 2 — Additional file 2: Interview guide mapped to the NIHBCC intervention fidelity checklist. [file 12998_2023_480_MOESM2_ESM.docx]

Additional file 2. Interview guide mapped to the NIHBCC intervention fidelity checklist

**Demographic questions:**

Profession: GP or Chiropractor

Location: Urban or Rural

Number of years in practice:

**Part 1** Discussion of intervention fidelity and the proposed intervention

**Presentation**

- Presentation on intervention fidelity (what it is and why it is important)
- Explain the aim of today’s interview
- Introduce the proposed intervention (clinical resource) and explain its goals

**Discussion**

| Questions | NIHBCC Intervention Fidelity Checklist (Borrelli 2005, 2011) |
| --- | --- |
| Do you think non-indicated imaging for LBP is an important issue?   - Follow up: Do you think an intervention to reduce non-indicated imaging for LBP is important? | Characteristics being sought in a treatment provider are articulated a priori (*Training*)  Assessment of whether or not there is a good fit between the provider and the intervention (*Training*) |
| What do you think about this clinical resource?   - Prompt: Is it good/bad? Will it be useful/not useful? | Assessment of whether or not there is a good fit between the provider and the intervention (*Training*) |

**Part 2** Fidelity of training

**Presentation**

- Present general strategies for enhancing and monitoring fidelity of *training*, as well as proposed strategies to be used in the study

**Discussion**

| Questions | NIHBCC Intervention Fidelity Checklist (Borrelli 2005, 2011) |
| --- | --- |
| What do you think about some of the potential strategies for ensuring intervention fidelity related to your training to use this clinical resource?   - Follow up: How do you feel they might work or not work for you? Why or why not? | There is a training plan that takes into account trainees’ different education and experience and learning styles |
| Do you think having a training manual will be helpful for your training?   - Follow up: What are some key things you would like to have in the training manual? What about formatting – paper or as a PDF? - Follow up: Are there other things you need to know/learn about in order to be properly trained in using the intervention? - Follow up: Are there other skills you think you need in order to be properly trained in the using the intervention? | Description of how providers will be trained (manual of training procedures)  Standardisation of provider training |
| Do you think regular booster sessions and/or support from the research team would be helpful? Why or why not?   - Follow up: What do you think would be the best way to provide support? | Monitoring of provider skill maintenance over time |
| What challenges can you see with respect to being able to attend the training session?  What resources would you need to help overcome these challenges?   - Prompt: location, time, in-person format | Not in checklist – Goal of question: Finding out what providers need in order to achieve high fidelity of training (e.g., being able to actually attend the sessions) |

**Part 3** Fidelity of intervention delivery

**Presentation**

- Present general strategies for enhancing and monitoring fidelity of *intervention delivery*, as well as proposed strategies to be used in the study

**Discussion**

| Questions | NIHBCC Intervention Fidelity Checklist (Borrelli 2005, 2011) |
| --- | --- |
| What do you think about some of the potential strategies for ensuring intervention fidelity related to using this clinical resource in your practice?   - Follow up: How do you feel they might work or not work for you? Why or why not? - Follow up: How do you feel about using audio recording to ensure adherence to protocol? | Method to ensure that the content of the intervention is delivered as specified  Mechanism to assess if the provider actually adhered to the intervention plan |
| Do you think having a script or manual for using the clinical resource will help you to better deliver the intervention to your patients? | Method to ensure that the content of the intervention is delivered as specified  Use of treatment manual |
| How easy or difficult do you think it would be to adhere to delivering >80% of the intervention components?   - Follow up: What aspects of the intervention seem easy? What ones seem more difficult to deliver? And why? - Follow up: What other things do you need to know/learn in order to deliver the intervention? - Follow up: What other skills do you need in order to deliver the intervention? | There is an a priori specification of treatment fidelity (e.g., providers adhere to delivering >80% of components) |
| What challenges related to resources can you see with respect to being able to deliver the intervention?   - Follow up: What do you need to help overcome these challenges? - Prompt: time, equipment, knowledge | Not in checklist – Goal of question: Finding out what providers need in order to achieve high fidelity of intervention delivery (e.g., being able to actually deliver the intervention) |
| What challenges related to patient factors do you think might influence your ability to deliver the intervention? | Not in checklist – Goal of question: Finding out what providers need in order to achieve high fidelity of intervention delivery (e.g., being able to actually deliver the intervention) |
| How important is it for you to deliver this intervention as planned?   - Follow up: What components of the intervention do you think needs to be more flexible/adaptable? | Not in checklist – Goal of question: For intervention planning |

Leading up to this interview, was there anything that you expected to talk about today that we didn’t discuss?

**Conclusion**

Could you please provide us (now or emailed to us at a later date) with two other practitioners who you think may be interested in participating in the study? We are looking for those from different geographical regions, as well as those who may have differing views or practice patterns to ensure a wide range of perspectives are captured in our study.
