# Supplementary material for: A qualitative study exploring perceived barriers and enablers to fidelity of training and delivery for an intervention to reduce non-indicated imaging for low back pain
Source: Chiropr Man Therap. 2023 Jan 31;31:6. doi: 10.1186/s12998-023-00480-6 (PMC9890790; doi:10.1186/s12998-023-00480-6)
Supplement: Supplementary file 3 — Additional file 3: Codebook for each domain in the Theoretical Domains Framework. [file 12998_2023_480_MOESM3_ESM.docx]

Additional file 3. Codebook for each domain in the Theoretical Domains Framework

**Target behaviour: Being trained in and delivering an intervention to reduce imaging for LBP with high fidelity**

| Domain | Definition, question, component constructs | Examples | Rationale |
| --- | --- | --- | --- |
| Knowledge | An awareness of the existence of something  **What do they know about imaging and LBP?**  **How does that influence their training in using the intervention?**  **How does that influence if they can/will deliver the intervention as planned?**   - Knowledge of condition/scientific rationale - Procedural knowledge - Knowledge of task environment | Q: In terms of your level of knowledge about imaging and back pain, where do you get your knowledge?  A: I think it was very much a fundamental part of our radiology training in school – to ensure that unnecessary imaging was not part of your clinical practice.  Q: Are you aware of clinical practice or professional guidelines for back pain?  A: Yes. We don’t have an official document, but that sentiment and that education will be reiterated regularly in our provided CE. There is a mandatory ongoing continuing education component for radiology. | Participant has knowledge about LBP and imaging. This can imply a good fit between the provider and the intervention. |
| Skills | An ability or proficiency acquired through practice  **What do they know about how to manage LBP without imaging?**  **How does that influence their training in using the intervention?**  **How does that influence if they can/will deliver the intervention as planned?**   - Skills - Skills development - Competence - Ability - Interpersonal skills - Practice - Skill assessment | I do find with patients when I take the time to explain to them that, not just from a radiation perspective, but that it won’t affect what we do, they’re very reasonable about [not having imaging for LBP]. I do think patients, when you take the time to actually educate them on it, they’re very receptive to not getting the image that they might have initially come to pursue at the beginning of the appointment. | Participant uses communication skills to educate the patient about LBP and imaging. This can imply a good fit between the provider and the intervention. |
| Social/professional role and identity | A coherent set of behaviours and displayed personal qualities of an individual in a social or  work setting  **Do they think it is their role as a healthcare provider and/or participant in the study to be adequately trained in how to use the intervention?**  **Do they think it is their role as a healthcare provider and/or participant in the study to deliver the intervention as planned?**   - Professional identity - Professional role - Social identity - Identity - Professional boundaries - Professional confidence - Group identity - Leadership - Organisational commitment | It’s always good to have your skills honed. It’s an uncomfortable thing, of course, to be taped, or especially at a certain point in your practice to feel you’re being evaluated in these things that you would like to think you’re quite proficient in. But none of us are proficient in everything every day. Hence, commitment to lifelong learning. I don’t think it’s a farfetched thing. I think it’s something that’s pretty fundamental to our daily practice.  I do feel that this stuff gives back to your whole profession and it gives back to your patient’s best interest. So, I think appealing to people in that regard would certainly invoke more of a commitment to doing it [participating in the study]. | Participant feels that it is their role to constantly review their skills.  Participant feels it is their role to participate in the study. |
| Beliefs about capabilities | Acceptance of the truth, reality, or validity **about an ability**, talent, or facility that a person can put  to constructive use  **How confident are they in being able to be trained in using the intervention?**  **How confident are they in delivering the intervention as planned?**  **Is it up to them whether they can be trained or deliver the intervention? (Perceived control)**   - Self-confidence - Perceived competence - Self-efficacy - Perceived behavioural control - Beliefs - Self-esteem - Empowerment - Professional confidence | I think it’s something we should be fairly comfortable in participating in to improve. | Participant has confidence in being able to deliver the intervention. |
| Optimism | The confidence that things will happen for the best or that desired goals will be attained  **How does whether they are optimistic/pessimistic influence their training in using the intervention?**  **How does whether they are optimistic/pessimistic influence whether they can/will deliver the intervention as intended?**   - Optimism - Pessimism - Unrealistic optimism - Identity | Q: Do you think this intervention would actually be helpful in reducing imaging by practitioners?  A: I do.  Q: What do you think about this specific clinical resource – do you think it’s good or bad?  A: I think it’ll be very good.  I think that a booklet of some sort that you can give to a patient would be very effective.  Q: Do you think people would not like to be audiotaped?  A: I think that people would buy into it. | Participant is optimistic about the intervention. |
| Beliefs about consequences | Acceptance of the truth, reality, or validity about outcomes of a behaviour in a given situation  **What will happen to them (their patients, their organisation, etc.) if they are trained in the intervention?**  **What will happen to them (their patients, their organisation, etc.) if they deliver the intervention as intended?**   - Beliefs - Outcome expectancies - Characteristics of outcome - expectancies - Anticipated regret - Consequents | It’s an uncomfortable thing, of course, to be taped.  I think that this would actually help to expedite an appointment in real practice. | Negative consequence of assessing fidelity (feeling uncomfortable).  Positive consequence of delivering the intervention as planned. |
| Reinforcement | Increasing the probability of a response by arranging a dependent relationship, or contingency, between the response and a given stimulus  **How will their previous experiences of managing LBP without imaging influence their training in using the intervention?**  **How will their previous experiences of managing LBP without imaging influence their ability to deliver the intervention as intended?**     - Rewards (proximal/distal, valued/not - valued, probable/improbable) - Incentives - Punishment - Consequents - Reinforcement - Contingencies - Sanctions | Q: If there was an incentive, it might be better?  A: Yeah, it is. | Being rewarded acts as a facilitator to training and delivery. |
| Intentions | A conscious decision to perform a behaviour or a resolve to act in a certain way  **Do they intend to/want to be trained in using the intervention?**  **Do they intend to/want to deliver the intervention as intended?**   - Stability of intentions - Stages of change model - Transtheoretical model and stages of change | I think that any type of booster session when you’re participating in something is good, you know, it keeps you accountable. And just in case you’re not exactly following protocols as set forth by the research study, then at least it can make sure that you’re participating as you had committed originally. | Participant wants to deliver the intervention as intended. |
| Goals | Mental representations of outcomes or end states that an individual wants to achieve  **Is being trained in using the intervention and managing LBP without imaging an important goal?**  **How much of a priority is their training in using the intervention compared to other competing demands?**  **Is delivering the intervention as intended and managing LBP without imaging an important goal?**  **How much of a priority is delivering the intervention as intended compared to other competing demands?**   - Goals (distal/proximal) - Goal priority - Goal/target setting - Goals (autonomous/controlled) - Action planning - Implementation intention | Q: Do you think non-indicated imaging for LBP is an important issue that needs to be addressed?  A: Very much.  Q: How important is it for you to deliver the intervention as planned?  A: Very important. | Participant thinks that imaging is an important issue. This can imply a good fit between the provider and the intervention.  Participant thinks it is important to deliver the intervention as planned. |
| Memory, attention and decision processes | The ability to retain information, focus selectively on aspects of the environment and choose  between two or more alternatives  **Will there be situations where they are likely to forget how they have been trained in using the intervention?**  **Will there be situations where they are likely to forget to deliver the intervention as intended?**  **Will there be situations where they decide not to deliver the intervention as intended?**     - Memory - Attention - Attention control - Decision making - Cognitive overload/tiredness | Would it be allowed if I had a patient come in and I just didn’t have time, so I skipped them? | Participant identifies a situation when they would not deliver the intervention as intended (when there is not enough time). |
| Environmental context and resources | Any circumstance of a person’s situation or environment that discourages or encourages the  development of skills and abilities, independence, social competence and adaptive behaviour  **What resources do they currently have that will allow them to be trained in using the intervention?**  **What resources do they currently have that will allow them to deliver the intervention as intended?**   - Environmental stressors - Resources/material resources - Organisational culture/climate - Salient events/critical incidents - Person × environment interaction - Barriers and facilitators | If I have them on a handout, it’s something that they’re receiving.  From a time perspective, that’s always the hardest thing in practice. I think you’ll probably get some resistance with respect to how long it would take for an appointment.  Q: What challenges might you see with respect to being able to attend the session?  A: I think for most, it would just be time. I would just have to schedule it in just like anything else. | Participant regularly uses handouts to help deliver information. This can imply that this practice will help them deliver the intervention as intended.  Participant identifies time as a barrier to delivering the intervention as intended.  Participant identifies time as a barrier to attending training for the intervention. |
| Social influences | Those interpersonal processes that can cause individuals to change their thoughts, feelings,  or behaviours  **Are they influenced by other people (e.g., patients, colleagues) to be trained in using the intervention?**  **Are they influenced by other people (e.g., patients, colleagues) to deliver the intervention as intended?**   - Social pressure - Social norms - Group conformity - Social comparisons - Group norms - Social support - Power - Intergroup conflict - Alienation - Group identity - Modelling | Q: What impact do you think an intervention like this will have on your patients?  A: Once they realise their education is in their best interest and not just because we can’t give it [imaging] to you, I think it’s very reasonable and I think people would be very receptive to it.  Q: For patients who really want to have imaging, do you think that would impact your ability to deliver this intervention?  A: No, I think it would be something to contend with, but I don’t think it would prevent me from doing it. | Participant thinks patients would be receptive. This could imply that they are more likely to deliver the intervention as intended.  Participant delivering the intervention as intended not influenced by patient requests for imaging. |
| Emotion | A complex reaction pattern, involving experiential, behavioural, and physiological  elements, by which the individual attempts to deal with a personally significant matter or event  **How do they feel about being trained in using the intervention and how do those feelings influence what they do (e.g., attending training)?**  **How do they feel about delivering the intervention as intended and how do those feelings influence what they do?**   - Fear - Anxiety - Affect - Stress - Depression - Positive/negative affect - Burn-out | I would probably opt for role playing nonetheless even though I’m not comfortable with it.  I think it [a script] would provide more comfort. I think instead of being seen as a time consumer, I think I’d feel that at least I was being thorough and that I wasn’t missing anything. I think that would be very comforting to know that you’re not missing anything. | Participant doesn’t feel comfortable with role play for training (barrier), but would still do it (enabler).  Participant feels comforted by having a script to help deliver the intervention as planned. |
| Behavioural regulation | Anything aimed at managing or changing objectively observed or measured actions  **What existing strategies do they have to support them in getting trained in using the intervention?**  **What additional strategies do they think would be helpful in getting trained in using the intervention?**  **What existing strategies do they have to support them in delivering the intervention as intended?**  **What additional strategies do they think would be helpful for them to deliver the intervention as intended?**   - Self-monitoring - Breaking habit - Action planning | I think if you attach CE hours to it [training], people will participate.  Q: What are some key things you would need to be in the training manual?  A: Algorithmic charts and visuals. Digital format because you can click on a link and it’s just tidy or you won’t lose it.  I think if the script could be honed enough that it would be all done in 15-20 minutes. It would be about fitting it into an appointment time. | Participant thinks CE credits would support getting clinicians trained in using the intervention.  Strategies to improve training in using the intervention.  Strategies to improve delivery of the intervention as planned. |
| Other codes (for inductive analysis) | Statements that do not directly relate to a domain but are relevant to the development of the intervention |  |  |
